# Technological applications to enhance independence in daily activities for older adults: a systematic review

**DOI:** 10.3389/fpubh.2024.1476916

**Published:** 2024-12-04

**Authors:** Carmen Requena, María Plaza-Carmona, Paula Álvarez-Merino, Verónica López-Fernández

**Affiliations:** ^1^Department of Psychology, University of León, León, Spain; ^2^Department of Education, University Internacional de La Rioja and León University Hospital Complex, León, Spain; ^3^Department of Social Work, University of Valladolid, Valladolid, Spain; ^4^Department of Neuropsychology, Universiad Internacional de La Rioja, Logroño, Spain

**Keywords:** independent older adults, sensor technologies, activities, technological taxonomy, technological development, systematic review

## Abstract

**Introduction:**

Monitoring daily activities in older adults using sensor technologies has grown significantly over the past two decades, evolving from simple tools to advanced systems that integrate Artificial Intelligence (AI) and the Internet of Things (IoT) for predictive monitoring. Despite these advances, there is still a need for a comprehensive review that addresses both technological progress and its impact on autonomous aging.

**Objective:**

To conduct a systematic review of sensor technologies used to monitor the daily activities of independent older adults, focusing on sensor types, applications, usage contexts, and their evolution over time.

**Methodology:**

A search was conducted in PubMed, Scopus, Web of Science, PsycInfo, and Google Scholar databases, covering studies published between 2000 and 2024. The 37 selected studies were assessed in terms of methodological quality and organized into four chronological stages, allowing for an examination of the progressive development of these technologies. Each stage represents an advance in sensor type, technological application, and implementation context, ranging from basic sensors to intelligent systems in multi-resident homes.

**Results:**

Findings indicate a clear progression in the accuracy and applicability of sensors, which evolved from fall detection to predictive interventions tailored to each user’s needs. Furthermore, the taxonomic classification of studies shows how sensors have been adapted to monitor physical, cognitive, and social dimensions, laying the groundwork for personalized care.

**Conclusion:**

Sensors represent a promising tool for promoting the independence and well-being of older adults, enabling proactive and personalized interventions in everyday settings. However, the lack of standardization in key parameters limits comparability between studies and highlights the need for consensus to facilitate the design of effective interventions that promote autonomous and healthy aging.

## 1 Introduction

Population aging is a global phenomenon that presents significant challenges for healthcare and welfare systems. The World Health Organization (WHO) estimates that by 2050, one in six people will be aged 60 or older, doubling the current proportion of older adults (1). This shift entails an increase in longevity as well as a growing demand for care models that prioritize autonomy and quality of life for older adults. In this context, the traditional association of old age with illness or dependency is gradually shifting, particularly as older adults reach advanced ages while maintaining high levels of functionality and well-being. Thus, the current “active aging” paradigm underscores the importance of maximizing opportunities for health, participation, and security, fostering a view of aging focused on personal development and independence (2).

Simultaneously, active aging requires tools that can support this comprehensive approach, assisting in health monitoring and management without compromising autonomy. Emerging technologies, particularly environmental sensors and wearable devices have shown great promise as solutions in this area (3). Initially, sensor use was limited to emergency detection and health event monitoring in highly dependent older adults, such as falls or dementia-related incidents. However, technological advancements have significantly expanded these applications, which now also benefit healthy and active older adults. Modern sensors enable continuous, non-invasive monitoring of physical, social, and cognitive activity parameters, providing data that enhances understanding of practices that contribute to the preservation of independence and quality of life in old age (4).

Furthermore, the integration of sensors with advanced technologies such as the Internet of Things (IoT) and Artificial Intelligence (AI) has further expanded their applications, enabling environmental monitoring at home in ways that adapt to the daily routines of older adults (5). These technologies capture data on daily activities, offering the opportunity for proactive assessment of changes in behavior and functional health. Unlike traditional monitoring models, which focus on reactive interventions, this approach allows for the anticipation of health issues and the customization of personalized interventions to meet the evolving needs of older adults (6, 7). By analyzing patterns of physical, social, and cognitive activity, AI can help identify practices and metrics associated with the preservation of functionality in autonomous older adults, thus optimizing preventive care approaches at this stage of life (8).

Within the framework of active aging, the use of these technologies provides benefits that go beyond health monitoring (9, 10). By capturing data that reflects the daily lives of older adults, these tools offer a holistic view of well-being, identifying practices that contribute to both physical health and personal development (11). The ability of these devices to analyze indicators of autonomy and adaptation is essential for promoting healthy and fulfilling aging. In particular, cognitive and social activities, such as interacting with others or engaging in intellectual pursuits, have been observed to play a crucial role in maintaining autonomy and reducing the risk of cognitive decline (12).

This study aimed to conduct a systematic review of sensor technologies used to monitor the daily activities of independent older adults, analyzing in depth not only the types of sensors used but also their applications, contexts of use, and specific objectives. Through a chronological organization in stages and an application taxonomy, this review provided a comprehensive view of the progress of these technologies, from basic detection tools to advanced predictive and personalized monitoring systems. This approach aimed to lay the groundwork for designing tailored interventions that promote independence and well-being in older adults, contributing to a better understanding of how these technologies can facilitate autonomous and healthy aging at home.

## 2 Methodology

We conducted a systematic review of scientific literature published between 2000 and 2024, along with a narrative synthesis of the selected studies. The search took place between May and October 2023 and followed four main phases: literature search, article selection, assessment of methodological quality, and data extraction and interpretation. Researchers evaluated the quality of the studies using specific checklists tailored to each study design: the STROBE checklist for observational studies (13), the CONSORT checklist for clinical trials (14), and the PRISMA statement for systematic reviews (15). The review included only studies that met a minimum percentage of relevant quality items on their respective checklists.

Data sources included PubMed, Scopus, Web of Science, PsycINFO, and Google Scholar. The search strategy was structured using the Boolean terms “AND” and “OR” to connect terms across two main topics. Search terms included combinations such as: (ag* OR old* OR older*) AND (“free-living” OR “community-dwelling” OR home) AND (“activities of daily living” OR ADL OR “personal development” OR growth OR “cognitive activities” OR “physical activity” OR “social activity” OR care* OR recognize*) AND (tech OR wearable OR sens OR device OR app OR smartphone OR “smart home” OR “human activity recognition” OR HAR OR “artificial intelligence” OR “deep learning” OR “neural network*” OR “supervised learning” OR “unsupervised learning”).

The inclusion criteria were: (a) studies evaluating activities of daily living (ADL), instrumental activities of daily living (IADL), and aspects of personal development (physical, cognitive, and social) in older adults; (b) studies that employed monitoring technologies or sensors to assess ADL, IADL, personal development, or safety in older adults; (c) studies focusing on older adults without health conditions that significantly impaired mobility or autonomy; and (d) studies conducted in everyday living environments (“free-living”) where participants resided in their own homes.

The exclusion criteria included: (a) studies exclusively focused on fall prevention or detection without evaluating other physical, cognitive, or social aspects, except for early studies (2000–2005) that, although centered on fall detection, contributed relevant technological advances for algorithm and sensor development; (b) studies dedicated solely to monitoring physical activity without considering other dimensions; (c) studies analyzing assistive technologies without continuous monitoring; (d) studies conducted only in experimental settings without real-life applications; and (e) studies published before 2000.

Four researchers initially screened the studies, and a fifth evaluator resolved any discrepancies. They reviewed titles and abstracts first, followed by a thorough evaluation of the full texts to confirm that each study met the inclusion criteria.

## 3 Results

**Figure 1:**
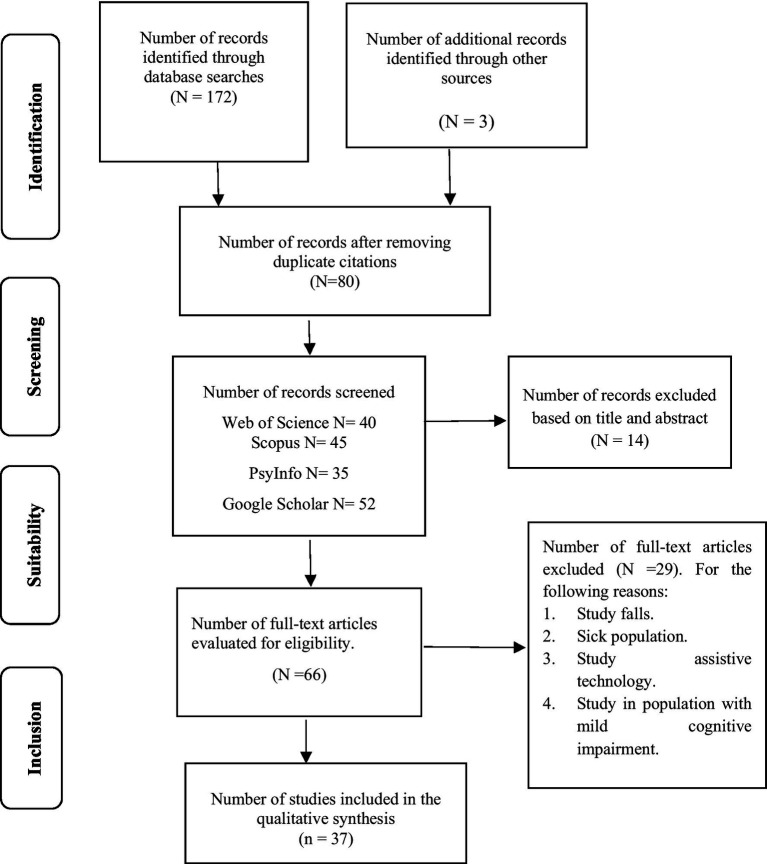
Flowchart of search process (PRISMA).

The systematic review followed the PRISMA method to ensure a thorough and transparent process for selecting and evaluating relevant literature. Following recommendations, we expanded the search, identifying a total of 172 documents from various sources. After removing 92 duplicates, 80 unique documents remained for initial evaluation. In the preselection phase, we excluded 14 articles that did not meet relevance or methodological quality standards. The remaining 66 documents underwent full review, and we excluded 29 of these due to methodological limitations or insufficient data. This process resulted in 37 studies that met all inclusion criteria and were incorporated into the qualitative synthesis (see Figure 1).

To facilitate analysis, we organized the 37 studies into four chronological stages, reflecting advancements in sensor technologies for monitoring older adults. We evaluated each study’s methodological quality based on sample size, bias control, follow-up duration, and reproducibility, using the STROBE and CONSORT criteria. Supplementary Tables S1, S2 provide further detail: one outlines the specific criteria for each methodological quality item, and the other presents a detailed evaluation of each study according to these criteria.

The review reveals a clear and continuous progression in sensor development and applications for older adults, structured into four chronological stages (see Table 1 in the main article). These advancements enabled sensors to evolve from basic fall detection tools in home environments to complex predictive and personalized monitoring systems in multi-resident communities, enhanced by AI.

**Table 1:** Summary of studies on sensor technologies for monitoring older adults.

Study (Year)	Title	Sensor type	Applications	Use context	Key technology	Sensor location	Measured parameters	Sampling frequency	Study duration
Stage 1: Initial Developments 2000–2005									
Orr & Abowd (2000) (20)	The smart floor: a mechanism for natural user identification and tracking	Contact Sensors	Identification and tracking	Experimental home setting	Smart floor	Floor	Ground Reaction Force (GRF)	93 Hz	Short (experimental)
Mathie et al. (2004) (22)	Accelerometry: providing an integrated, practical method for long-term, ambulatory monitoring of human movement	Accelerometers	Fall monitoring	Home	Waist device	Waist	3-axis acceleration	20 Hz	1 week
Sixsmith & Johnson (2004) (21)	A smart sensor to detect the falls of the older adult	Infrared sensors	Fall detection	Home and care homes	Low-cost infrared sensors	Not body-specific	Thermal movement	Variable	Several months (field tests)
Tapia et al. (2004) (23)	Activity recognition in the home using simple and ubiquitous sensors	State sensors	Daily activity recognition	Home	Low-cost sensors	Household objects	Change status (ON/OFF)	N/A	2 weeks
Stage 2: Advances in Wearables and Remote Monitoring (2006–2010)									
Alemdar & Ersoy (2010) (24)	Wireless sensor networks for healthcare: A survey	Wireless sensor networks	Remote health and activity monitoring	Smart home	Wireless networks	N/A	Activity frequency	N/A	Variable (surveys)
Bourke et al. (2007) (17)	Evaluation of a threshold-based tri-axial accelerometer fall detection algorithm	Accelerometers	Fall detection	Home	Tri-axial accelerometer	Waist and thigh	Acceleration and orientation	100 Hz	Several days (simulation)
Hayes et al. (2008) (16)	Unobtrusive assessment of activity patterns associated with mild cognitive impairment	Motion sensors	Cognitive impairment assessment	Home	Environmental monitoring	N/A	Gait speed	24/7 continuous	Average 315 days
Kangas et al. (2008) (19)	Comparison of low-complexity fall detection algorithms for body attached accelerometers	Accelerometers	Fall Detection, Daily Activity Levels	Home	Low-Complexity Algorithms	Waist and Head	Resultant Acceleration, step Count	400 Hz	1 day (simulation)
Chan et al. (2009) (25)	Smart homes - Current features and future perspectives	Motion Sensors	Behavior Monitoring	Smart Home	Environmental Monitoring	Home	Movement Activity	24/7 Continuous	Variable
Hagler et al. (2010) (26)	An unobtrusive method for monitoring in-home walking speed	Environmental Sensors	Gait Speed Monitoring	Smart Home	Motion Sensors	Home	Gait Speed	N/A	6 months
Virone et al. (2008) (27)	Behavioral patterns of older adults in assisted living	Contact and Motion Sensors	Health and Daily Activity Monitoring	Smart Home	Contact and Motion Sensors	No specific location	Activity Frequency and Presence	N/A	Variable
Zouba et al. (2010) (28)	An activity monitoring system for real older adult at home: Validation study	Motion Sensors and Cameras	Daily Activity and Fall Monitoring	Smart Home	Sensor and Camera Network	No specific location	Home Activity and Movement	24/7 continuous	Variable
Stage 3. Expansion of Applications and Enhancement of Autonomy (2011–2015)									
Kaye et al. (2011) (4)	Intelligent systems for assessing aging changes: home-based, unobtrusive, and continuous assessment of aging	Environmental sensors	Continuous aging assessment	Assisted living	Continuous environmental monitoring	No specific location	Movements, time at home	24/7 continuous	Average 33 months
Dawadi et al. (2013) (29)	Automated assessment of cognitive health using smart homes	Environmental sensors, AI	Cognitive health assessment	Smart home	Sensors and AI algorithms	Not body-specific	Cognitive activity quality	Variable	Variable (temporal analysis)
Fleury et al. (2010) (30)	SVM-based multimodal classification of activities of daily living in health smart homes: Sensors, algorithms, and first experimental results	Motion sensors	Daily activity recognition	Home	Motion sensors |	Waist	Movement patterns	50 Hz	1 month
Nef et al. (2015) (31)	Evaluation of Three State-of-the-Art Classifiers for Recognition of Activities of Daily Living from Smart Home Ambient Data	Environmental sensors	Daily activity recognition	Smart home	ADL classification	Not body-specific	Activities of daily living	Variable	200 days
Dasios et al. (2015) (32)	Hands-on experiences in deploying cost-effective ambient-assisted living systems	Accelerometers	Activity and fall recognition	Residential setting	Body-worn accelerometers	Wrist, ankle	Acceleration	100 Hz	7 days
Ni et al. (2015) (33)	The Elderly’s Independent Living in Smart Homes: A Characterization of Activities and Sensing Infrastructure	Environmental and contact sensors	Daily activity monitoring	Smart home	Environment-based sensors	Household objects	Usage status (ON/OFF)	N/A	Variable
Stage 4. Advanced Integration of AI (2016–2024)									
García-Moreno et al. (2020) (34)	A microservices e-Health system for ecological frailty assessment using wearables	Health sensors (IoT)	Frailty assessment	Smart home	Frailty microservices	Wrist, waist	Frailty	Variable	3 months
Muangprathub et al. (2021) (35)	A novel older adult tracking system using machine learning	Motion sensors	Activity tracking with AI	Smart home	Applied artificial intelligence	N/A	Activity patterns	50 Hz	30 days
Debes et al. (2016) (36)	Monitoring activities of daily living in smart homes	Motion and contact sensors	Daily activity monitoring	Smart home	Behavior sensors	Not body-specific	Daily living activities	Variable	Variable
Moschetti et al. (2016) (37)	No disponible en vista previa	Motion and contact sensors	Daily activity monitoring	Smart home	Behavioral sensors	No specific location	Daily activity	Variable	Variable
Schrack et al. (2018) (38)	Using Heart Rate and Accelerometry to Define Quantity and Intensity of Physical Activity in Older Adults	Environmental and health sensors	Wellness management	Smart home	IoT health sensors	Blood pressure, heart rate	24/7 continuous	24/7 continuo	1 year
Sepesy et al. (2021) (39)	Discovering daily activity patterns from sensor data sequences and activity sequences	Motion sensors	Daily activity recognition	Smart home	Machine learning	**Wrist**	Acceleration	60 Hz	2 weeks
Schrack et al. (2016) (40)	Assessing Daily Physical Activity in Older Adults: Unraveling the Complexity of Monitors, Measures, and Methods	Environmental sensors	Probabilistic ADL recognition	Home	Probabilistic models	No specific location	ADL patterns	Variable	3 months
Vervoort et al. (2016) (41)	Multivariate analyses and classification of inertial sensor data to identify aging effects on the timed-up-and-go test	Wearable sensors	Health and mobility monitoring	Residential settings	Wearables on the body	Wrist, ankle	Acceleration and orientation	100 Hz	1 month
Gomez-Ramos et al. (2021) (42)	Daily human activity recognition using non-intrusive sensors	Environmental and contact sensors	Cognitive function assessment	Smart home	Cognitive monitoring	No specific location	Changes in usage patterns	N/A	4 months
Igarashi et al. (2022) (43)	Eliciting a User’s Preferences by the Self-Disclosure of Socially Assistive Robots in Local Households of Older Adults to Facilitate Verbal Human–Robot Interaction	Environmental and status sensors	Activity recognition	Home	Low-cost sensors	Household items	Status (ON/OFF)	N/A	2 months
Papagiannaki et al. (2019) (44)	Recognizing physical activity of older people from wearable sensors and inconsistent data	AI sensors	Real-time monitoring	Home	Artificial intelligence	N/A	Activity and health	24/7 continuous	1 year
Sasaki et al. (2016) (45)	Performance of Activity Classification Algorithms in Free-living Older Adults	Accelerometers (ActiGraph GT3X+)	Activity type classification	Laboratory and free-living environment	Machine learning algorithms (SVM and Random Forest)	Wrist, hip, and ankle	Activity types (sedentary, locomotion, domestic)	80 Hz	2–3 h in free-living environment
Naccarelli et al. (2022) (6)	The Problem of Monitoring Activities of Older People in Multi-Resident Scenarios: An Innovative and Non-Invasive Measurement System Based on Wearables and PIR Sensors	Motion and environmental sensors	Assistance in daily living	Home	Home automation	Home	Activity and time at home	24/7 continuous	1 year
Genovese et al. (2017) (46)	A smartwatch step counter for slow and intermittent ambulation	Contact and environmental sensors	Health monitoring at home	Smart home	Connected sensors	No specific location	Blood pressure, glucose, activity	Variable	Variable
Paraschiakos et al. (2020) (47)	Activity recognition using wearable sensors for tracking the older adult	Fusion sensors	Health monitoring and attention	Smart home	IoT data fusion	No specific location	Variability in patterns	24/7 continuous	9 months
Rejeski et al. (2016) (48)	Analysis and Interpretation of Accelerometry Data in Older Adults: The LIFE Study	Accelerometers (ActiGraph GT3X)	Physical activity assessment	Daily activity in free-living and controlled contexts	Accelerometry for activity monitoring	Right hip	Counts of activity (CPM), exercise intensity	30 Hz	7 days of continuous monitoring
Aramendi et al. (2018) (49)	Automatic assessment of functional health decline in older adults based on smart home data	Motion sensors	Recognition in independent living	Residences	Motion sensors	Waist, ankle	Acceleration	40 Hz	6 weeks
Gochoo et al. (2017) (50)	DCNN-based older adult activity recognition using binary sensors	Motion sensors	Activity recognition	Home	Predictive algorithms	No specific location	Daily activity levels, Interaction pattern	Variable	1 year
Bianchi et al. (2019) (51)	RSSI-Based Indoor Localization and Identification for ZigBee Wireless Sensor Networks in Smart Homes	AI and environmental sensors	Comprehensive health monitoring	Smart home	Artificial intelligence and environmental sensors	No specific location	Health patterns	24/7 continuous	1 year

The focus and key technologies of each stage are detailed below:

Stage 1 (2000–2005): Early studies employed contact sensors and basic accelerometers, primarily focusing on fall detection and simple activity monitoring. This phase was crucial for testing sensor feasibility with older adults, enabling the capture of movement patterns and specific events. Although some studies, such as Kangas et al. (19), focused solely on fall detection, their inclusion in this review is justified by their contribution to early algorithm and sensor technology development, laying the foundation for continuous monitoring in everyday contexts.

Stage 2 (2006–2010): In this stage, the use of triaxial accelerometers and wireless networks advanced remote monitoring, allowing for applications like activity pattern and gait speed assessment. Sensors began capturing real-time information in smart homes, supporting immediate interventions for falls or mobility changes.

Stage 3 (2011–2015): This period saw the integration of advanced environmental sensors and combined accelerometers and gyroscopes, enabling applications focused on maintaining independence in daily activities and monitoring cognitive health. These systems supported continuous, detailed monitoring in homes and assisted living environments, allowing for the detection of changes in functionality and cognition among older adults.

Stage 4 (2016–2024): In this most recent stage, IoT and AI technologies expanded monitoring scope, emphasizing prediction and personalization. Studies in this period utilized advanced devices to detect and prevent falls and conduct predictive analyses on functional decline. Monitoring also extended to social aspects, capturing interaction patterns in multi-resident settings and enabling proactive, personalized support for older adults.

Table 1 summarizes these advancements across three key dimensions:

Type of Sensor: Progressing from contact sensors and basic accelerometers in Stage 1 to advanced IoT and AI devices in Stage 4.

Technological Applications: Evolving from fall detection in Stage 1 to predictive analysis and social monitoring in Stage 4.

Context of Use: Shifting from home environments in Stage 1 to multi-resident communities in Stage 4, allowing for adaptive and continuous monitoring.

Table 2 presents a taxonomy of sensor use in older adults, organized into three dimensions: population profile, monitoring objectives, and technological configuration. This structure illustrates how sensor applications adapt to different health contexts and specific population needs:

1. Target Population: Classification of studies based on healthy older adults, those with specific conditions (such as cognitive or mobility limitations), and groups in various settings (homes, assisted living facilities, hospitals).
2. Purpose of Use: Monitoring objectives, including daily activities (ADLs and IADLs), fall detection, cognitive and social well-being, and the management of specific health conditions.
3. Implementation Method: Details the technological configuration of sensors, specifying placement (such as wrist or environment), type of technology (wearables or environmental sensors), and sampling frequency.

**Table 2:** Taxonomy of sensor applications in older adults.

Use of sensors	Subcategory	Description	Type of activity	Definition and operationalization	Studies
Who Are They Applied To	Healthy Older Adults	Studies focused on older adults without specific conditions, mainly to assess their activity and well-being.	Physical Activity	Monitored activities include walking, sitting, and standing, using accelerometers to quantify movement patterns.	(20–23, 25, 26, 37, 40, 46, 48)
	Older Adults with Specific Conditions	Studies targeting older adults with mobility issues, cognitive decline, and other specific health needs.	ADL, Physical Activity	Activities include assisted walking, limited movements, and fall detection, assessed using specific motion sensors.	(4, 16, 17, 19, 27, 28, 31, 34, 44, 47)
	Specific Groups in Different Environments	Studies in settings such as residences, private homes, or hospital environments, adapted to the context of the place.	ADL, Social Activity	Monitored activities adjust based on the environment, such as mobility in common areas and use of specific spaces (e.g., bathroom, dining area).	(6, 30, 32, 33, 36, 39, 41–43, 51)
For What They Have Been Used	Monitoring Daily Activities (ADLs and IADLs)	Assessment of basic and advanced activities to promote independence and quality of life.	ADL, IADL	Monitored activities include dressing, eating, and personal hygiene (ADLs), as well as advanced activities like cooking or managing finances (IADLs), using contact and motion sensors.	(21, 30–33, 36, 37, 43, 49)
	Fall Detection and Mobility Risks	Use of sensors for detecting falls and risky mobility patterns, allowing for quick interventions.	Physical Activity	Falls are detected using accelerometers and gyroscopes that record sudden changes in movement and loss of balance.	(17, 19, 22, 28, 44, 45, 47)
	Cognitive and Social Well-Being Monitoring	Studies focused on mental health and social well-being, monitoring cognitive functions and social interactions.	Cognitive, Social Activity	Activities include time spent in social interaction, frequency of visits, and recognition of patterns in daily interactions.	(29, 34, 38, 42, 48, 50)
	Monitoring and Management of Specific Conditions	Monitoring particular health issues, such as symptom control and mobility assessment.	Physical Activity, ADL	Monitored activities include symptom control (e.g., tremors, posture changes) and movements within the home.	(4, 6, 16, 26, 39, 41, 46, 51)
How They Have Been Implemented	Sensor Location	Specific location of sensors, such as wrist, waist, ankle, or in the home, affects the type of data collected.	–	Sensors on the wrist and ankle capture specific movements such as walking, lifting arms, or bending knees.	(17, 19, 20, 22, 26, 41, 44–47)
	Type of Technology	Difference between wearables (accelerometers on wrist) and environmental sensors (contact, motion) in the home.	–	Use of accelerometers for monitoring continuous physical activity; contact sensors in smart floors to detect falls.	(6, 27, 37, 38, 40, 48, 50, 51)
	Sampling Frequency and Configuration	Configurations such as frequency in Hz and whether monitoring is continuous or intermittent, adjusted according to the application.	–	Sampling frequency varies (30–60 Hz) depending on the type of activity to monitor, such as rapid movement in falls or continuous activity in ADLs.	(24, 25, 32, 34, 41–43)

This taxonomy provides a clear and organized reference that facilitates the identification of sensor applications across various daily life contexts, helping to understand how technology can adapt to the needs and characteristics of different groups of older adults.

## 4 Discussion

The progression of sensor technology in monitoring older adults, organized into stages, demonstrates significant progress from basic devices to advanced technologies incorporating AI and IoT. This progression reflects not only improvements in the accuracy and reach of these technologies but also a shift toward a more comprehensive, personalized approach that encompasses the physical, cognitive, and social dimensions of aging. Recent studies underscore the importance of these advancements, showing that integrating sensors into everyday life can improve quality of life and support autonomy in aging (16).

The taxonomic classification presented in this review offers a new perspective by structuring sensor use according to ‘the target population,’ ‘purposes,’ and ‘implementation methods.’ This approach facilitates the grouping of studies according to specific monitoring objectives, older adults’ profiles, and application contexts, enabling the identification of usage patterns and highlighting areas for future research. Although early studies focused on older adults without complex conditions, the evolution of sensors has enabled greater personalization, adapting to subgroups with special needs, such as those with cognitive impairment or reduced mobility, and providing more in-depth monitoring oriented toward early intervention (17, 18).

From a practical standpoint, monitoring cognitive and social aspects represents an advance toward a holistic approach to health, going beyond an exclusively physical focus. Currently, researchers often infer cognitive activity indirectly, typically from movement patterns or social interactions (19, 20). Incorporating direct metrics on these activities would allow researchers and clinicians to gain a more detailed view of how cognitive and reflective practices contribute to personal development and healthy aging. Activities such as introspection and self-analysis have shown positive impacts on self-perception and adaptability in later life, which are associated with successful aging (21).

The variability in sensor configurations across studies also underscores the need to establish standards for parameters such as sampling frequency, device placement, and monitoring duration. Recent studies indicate that a lack of uniformity in these aspects limits the comparability and generalizability of findings (16, 17). In this regard, the adoption of uniform guidelines would not only improve the consistency of results but would also facilitate the application of these technologies in both clinical and everyday settings, allowing for more effective and accessible integration into health and social care systems for older people.

The progression of AI and IoT integration allows unprecedented levels of personalization in monitoring and adapting to real-time changes in older adults’ health status. This advancement holds significant implications for the future of healthcare, as it enables preventive interventions before critical events occur. Predictive capabilities are essential in the context of aging, allowing proactive planning that reduces reliance on intensive medical services and enhances the quality of life (12). However, using AI in monitoring also raises ethical and privacy concerns that must be addressed carefully, especially in multi-resident environments (18).

## 5 Limitations and recommendations for future research

This review, while comprehensive, has certain limitations inherent to our methodology and approach. First, our reliance on studies published primarily in English and from the year 2000 onward may introduce selection bias, potentially excluding relevant research in other languages or earlier work that could offer additional historical context on sensor technology in aging populations (15).

Second, there is considerable variability in study design and reporting across the selected literature, especially regarding sensor placement, sampling frequencies, and data processing methods. These methodological inconsistencies limit the comparability of findings and the ability to generalize conclusions across studies. Standardizing parameters such as sensor placement and data processing would enhance data consistency, facilitating broader application of results in clinical and research settings (16, 17).

Third, given the rapid evolution of sensor technology, particularly with advances in Artificial Intelligence (AI) and the Internet of Things (IoT), some findings from this review may become quickly outdated, impacting the long-term relevance of our conclusions (18).

Lastly, while we aimed to capture a balanced view of applications in physical, cognitive, and social monitoring, the existing literature tends to emphasize physical activity monitoring, with less focus on cognitive and social activities. Although some studies infer cognitive and social engagement indirectly, the limited direct monitoring of these domains restricts our ability to fully assess their impact on functional independence and quality of life in older adults (4, 6).

## 6 Conclusion

In conclusion, combining chronological and taxonomic perspectives in this review provides a comprehensive understanding of sensor use in older adults. This analysis highlights the technological advancements achieved, as well as the pressing need to establish standards and ethical frameworks that maximize their utility and accessibility in both clinical and everyday applications. Sensor-based monitoring, tailored for the physical, cognitive, and social needs of older adults, presents a significant opportunity to transform older adults care, fostering truly autonomous, healthy, and enriching aging.

While this systematic review demonstrates clear progress in the use of sensors to promote autonomy and healthy aging, challenges remain that continue to limit their long-term implementation and effectiveness. The lack of consensus on technical standards regarding sensor configuration, placement, and data interpretation hinders the creation of a unified framework that allows meaningful comparisons across studies. Furthermore, reliance on indirect methods for assessing cognitive and reflective activities restricts the scope of data obtained, underscoring the need for innovations that capture these essential aspects of aging more directly and in greater detail.

Overall, the findings of this review emphasize the potential of sensors to transform monitoring in older adults into tools for proactive and personalized intervention. Achieving greater standardization and developing technologies that address cognitive and reflective dimensions of aging will represent essential steps toward fully harnessing these tools. As these advancements become more established, sensor use could offer an increasingly effective strategy to foster autonomous, resilient, and meaningful aging, aligned with the growing demands of aging societies.

## Data Availability

The original contributions presented in the study are included in the article/Supplementary material, further inquiries can be directed to the corresponding author.
