# Supplementary material for: Technological applications to enhance independence in daily activities for older adults: a systematic review
Source: Front Public Health. 2024 Dec 4;12:1476916. doi: 10.3389/fpubh.2024.1476916 (PMC11652366; doi:10.3389/fpubh.2024.1476916)
Supplement: Supplementary file 1 [file Table_1.docx]

Supplementary Material

Table S1. Summary of Methodological Quality of Sensor Studies in Older Adults According to STROBE and CONSOR.

| **Evaluation Variable** | **Criterion** | **Description** | **Score** |
| --- | --- | --- | --- |
| Quality Score | Full Compliance | Meets 90-100% of the items on the corresponding checklist | 9-10 points |
|  | High Compliance | Meets 70-89% of the items | 7-8 points |
|  | Moderate Compliance | Meets 50-69% of the items | 5-6 points |
|  | Low Compliance | Meets 30-49% of the items | 3-4 points |
|  | Very Low Compliance | Meets less than 30% of the items | 1-2 points |
| Representative Sample | Adequate | Sample size >100 in observational studies, >30 per group in trials | Yes |
|  | Partial | Sample size between 50-100 in observational studies, 15-30 per group in trials | Partial |
|  | Inadequate | Sample size <50 in observational studies, <15 per group in trials | No |
| Bias Control | Complete | Use of rigorous randomization and blinding methods, as applicable | Yes |
|  | Partial | Some bias control, but not comprehensive | Partial |
|  | Absent | No clear bias control measures | No |
| Follow-up Period | Long | >6 months | Long |
|  | Adequate | 3-6 months | Adequate |
|  | Short | <3 months | Short |
| Reproducibility | High | Detailed and transparent methodology | Yes |
|  | Moderate | Some clear methodological details, but lacks specificity in certain procedures | Partial |
|  | Low | Insufficiently detailed methodology, making replication difficult | No |
| Observations/Limitations | - | Specific description of study limitations (e.g., small sample, short duration) | Descriptive text |

**Table S2**. Methodological Quality of Studies on Sensors for Monitoring Activities in Older Adults

| **Study (Authors, Year)** | **Study Design** | **Checklist Type** | **Quality Score** | **Sample Size (N)** | **Representative Sample** | **Bias Control** | **Follow-up Period** | **Reproducibility** | **Observations/**  **Limitations** | **Checklist** |
| --- | --- | --- | --- | --- | --- | --- | --- | --- | --- | --- |
| **STAGE 1. Initial Developments 2000 to 2005** | | | | | | | | | | |
| Orr & Abowd, 2000 | Observational | STROBE | 6 | 25 | No | Partial | Short | Partial | Small sample for generalization | 6 |
| Mathie et al., 2004 | Clinical Trial | CONSORT | 8 | 50 | Yes | Complete | Adequate | Yes | Well-defined methodology | 8 |
| Sixsmith & Johnson, 2004 | Observational | STROBE | 7 | 60 | Yes | Partial | Short | Yes | Limitations in follow-up | 7 |
| Tapia et al., 2004 | Observational | STROBE | 5 | 30 | No | No | Short | No | Lack of variable control | 5 |
| **STAGE 2. Advances in Wearables and Remote Monitoring (2006-2010)** | | | | | | | | | | |
| Alemdar et al., 2010 | Observational | STROBE | 8 | 100 | Yes | Complete | Long | Yes | Good data tracking and analysis | 8 |
| Bourke et al., 2007 | Clinical Trial | CONSORT | 7 | 40 | Yes | Partial | Adequate | Yes | Lack of blinding in part of the study | 7 |
| Hayes et al., 2008 | Clinical Trial | CONSORT | 6 | 60 | No | Partial | Adequate | Partial | Small sample and limited follow-up | 6 |
| Kangas et al., 2008 | Observational | STROBE | 8 | 120 | Yes | Complete | Long | Yes | Good design and detailed analysis | 8 |
| Chan et al., 2009 | Observational | STROBE | 6 | 75 | Partial | Partial | Short | Partial | Limitations in sample diversity | 6 |
| Hagler et al., 2010 | Observational | STROBE | 9 | 150 | Yes | Complete | Long | Yes | Excellent validity and adequate follow-up | 9 |
| Virone et al., 2008 | Observational | STROBE | 5 | 30 | No | No | Short | No | Few bias control measures | 5 |
| Zouba et al., 2010 | Observational | STROBE | 7 | 90 | Yes | Partial | Adequate | Yes | Limitations in follow-up duration | 7 |
| **STAGE 3. Expansion of Applications and Improvement of Autonomy (2011-2015)** | | | | | | | | | | |
| Kaye et al., 2011 | Observational | STROBE | 9 | 140 | Yes | Complete | Long | Yes | Excellent methodology and data analysis | 9 |
| Dawadi et al., 2013 | Observational | STROBE | 7 | 90 | Partial | Partial | Adequate | Yes | Limitations in sample diversity | 7 |
| Fleury et al., 2010 | Clinical Trial | CONSORT | 6 | 50 | No | Partial | Short | Partial | Small sample and inadequate follow-up | 6 |
| Nef et al., 2015 | Clinical Trial | CONSORT | 8 | 60 | Yes | Complete | Adequate | Yes | Good bias control | 8 |
| Dasios et al., 2015 | Observational | STROBE | 8 | 110 | Yes | Complete | Long | Yes | Good analysis and data robustness | 8 |
| Ni et al., 2015 | Observational | STROBE | 5 | 30 | No | No | Short | No | Small sample and lack of bias control | 5 |
| **STAGE 4. Advanced Integration of AI (2016-2024)** | | | | | | | | | | |
| Moreno et al., 2020 | Clinical Trial | CONSORT | 8 | 100 | Yes | Complete | 12 weeks | Yes | Good follow-up | 8 |
| Muangprathub et al., 2021 | Observational | STROBE | 7 | 60 | Yes | Complete | 30 days | Yes | Robust analysis | 7 |
| Debes et al., 2016 | Observational | STROBE | 6 | 100 | Yes | Complete | 84 days | Yes | Good bias control | 6 |
| Moschetti et al., 2016 | Observational | STROBE | 6 | 55 | No | No | 14 days | No | Small sample and lack of control | 6 |
| Schrack et al., 2018 | Observational | STROBE | 9 | 85 | Yes | Complete | 30 days | Yes | Excellent methodology | 9 |
| Sepesy Maučec et al., 2021 | Observational | STROBE | 8 | 70 | Yes | Complete | 90 days | Yes | Good data analysis | 8 |
| Schrack et al., 2016 | Observational | STROBE | 9 | 100 | Yes | Complete | \|  \| \| --- \|   3 months | Yes | Complexity of monitors, detailed methodology | 9 |
| Vervoort et al., 2016 | Observational | STROBE | 6 | 90 | No | Partial | 30 days | No | Lack of bias control | 6 |
| Gómez Ramos et al., 2021 | Observational | STROBE | 7 | 70 | No | Partial | 30 days | No | Non-representative sample | 7 |
| Igarashi et al., 2020 | Observational | STROBE | 7 | 50 | Yes | Complete | 30 days | Yes | Good study design | 7 |
| Papagiannaki et al., 2019 | Observational | STROBE | 5 | 40 | No | No | 10 days | No | Small sample and lack of control | 5 |
| Sasaki et al., 2016 | Observational | STROBE | 7 | 100 | Yes | Complete | 7 days | Yes | Good data control | 7 |
| Naccarelli et al., 2022 | Observational | STROBE | 8 | 120 | Yes | Complete | 60 days | Yes | Good variable control | 8 |
| Genivese et al., 2021 | Observational | STROBE | 8 | 90 | Yes | Complete | 14 days | Yes | Comprehensive analysis | 8 |
| Paraschakos et al., 2020 | Clinical Trial | CONSORT | 9 | 65 | Yes | Complete | 30 days | Yes | Robust design and good metrics | 9 |
| Rejesk et al., 2021 | Observational | STROBE | 6 | 30 | No | No | 14 days | No | Population limitations | 6 |
| Rejesk et al., 2021 | Observational | STROBE | 6 | 30 | No | No | 14 days | No | Population limitations | 6 |
| Aramendi et al., 2018 | Observational | STROBE | 8 | 90 | Yes | Complete | 7 days | Yes | Robust analysis | 8 |
| Gacho et al., 2019 | Observational | STROBE | 9 | 150 | Yes | Complete | 30 days | Yes | Excellent methodology | 9 |
| Bianchi et al., 2021 | Observational | STROBE | 7 | 60 | Yes | Partial | 30 days | Yes | Limitations in Follow-up | 7 |
